# RcsAB and Fur Coregulate the Iron-Acquisition System via *entC* in *Klebsiella pneumoniae* NTUH-K2044 in Response to Iron Availability

**DOI:** 10.3389/fcimb.2020.00282

**Published:** 2020-06-10

**Authors:** Lingyue Yuan, Xuan Li, Ling Du, Kewen Su, Jiaxue Zhang, Pin Liu, Qiang He, Zhongshuang Zhang, Dan Peng, Lifei Shen, Jingfu Qiu, Yingli Li

**Affiliations:** ^1^School of Public Health and Management, Chongqing Medical University, Chongqing, China; ^2^Hangzhou Hospital for the Prevention and Treatment of Occupational Disease, Hangzhou, China; ^3^Nanjing Center for Disease Control and Prevention, Nanjing, China

**Keywords:** RcsAB, Fur, *entC*, iron-acquisition system, *Klebsiella pneumoniae*

## Abstract

The iron acquisition system is an essential virulence factor for human infection and is under tight regulatory control in a variety of pathogens. Ferric-uptake regulator (Fur) is one of Fe^2+^-responsive transcription factor that maintains iron homeostasis, and the regulator of capsule synthesis (Rcs) is known to regulate exopolysaccharide biosynthesis. We speculate the Rcs may involve in iron-acquisition given the identified regulator box in the upstream of *entC* that participated in the biosynthesis of enterobactin. To study the coregulation by RcsAB and Fur of *entC*, we measured the β-galactosidase activity and relative mRNA expression of *entC* in WT and mutant strains. The RcsAB- and Fur-protected regions were identified by an electrophoretic mobility shift assay (EMSA) and a DNase I footprinting assay. A regulatory cascade was identified with which Fur repressed *rcsA* expression and reduced RcsAB and *entC* expression. Our study demonstrated that *entC* was coregulated by two different transcriptional regulators, namely, RcsAB and Fur, in response to iron availability in *Klebsiella pneumoniae*.

## Introduction

*Klebsiella pneumoniae* is an opportunistic pathogen that causes severe infections, mainly manifesting as pneumonia, bacteremia, septicemia, and urinary and respiratory tract infections (Podschun and Ullmann, 1998). A number of virulence factors identified in *K. pneumoniae* are involved in pathogenicity, including capsule polysaccharide (CPS), lipopolysaccarides, type 1 and 3 fimbriae, biofilm formation-related factors, urease an the iron-acquisition system (Clegg and Murphy, 2016; Paczosa and Mecsas, 2016; Lam et al., 2018; Bengoechea and Sa Pessoa, 2019; Russo and Marr, 2019).

The regulator of capsule synthesis (Rcs) phosphorelay is a complex signal transduction pathway composed of RcsB, RcsC, and RcsD (Majdalani and Gottesman, 2005; Wall et al., 2018). RcsC, a transmembrane sensor kinase, transfers a phosphoryl group to another membrane-spanning protein, RcsD, and finally to the response regulator, RcsB (Clarke, 2010). In addition to acting alone as a transcriptional regulators (Casino et al., 2018; Filippova et al., 2018), RcsB can also combine with the accessory protein RcsA to regulate related genes (Mouslim et al., 2003; Liu et al., 2014; Fang et al., 2015; Su et al., 2018). The Rcs phosphorelay plays a major role in the regulation of CPS (Mouslim et al., 2003; Llobet et al., 2011; Pando et al., 2017; Peng et al., 2018; Walker et al., 2019), biofilm formation (Sun et al., 2012; Fang et al., 2015), flagellar biogenesis (Lehti et al., 2012; Jozwick et al., 2019). The RcsAB binding site (RcsAB box) consists of a 7-7 inverted repeat sequence, TAAGAAT-ATTCTTA (Fang et al., 2015). The promoter-proximal regions of *entC* contain a RcsAB box-like sequence, so we hypothesize that the expression of *entC* is regulated by RcsAB.

There are 10 putative iron uptake systems in *K. pneumoniae* strain NTUH-K2044. Among those, 4 are siderophore-dependent, namely enterobactin (*entABCDEF*), yersiniabactin (*Yersinia* HPI), aerobactin (*iucABCDiutA*), and salmochelin (*iroNDCB*) (Hsieh et al., 2008). Siderophore is considered to be an important iron acquiring strategy by *K. pneumoniae*, especially under iron-restricted conditions (Paczosa and Mecsas, 2016). While yersiniabactin, aerobactin, and salmochelin predominate in pyogenic live abcess-associated *K. pneumoniae* strains (Hsieh et al., 2008), enterobactin is ubiquitous among almost all *K. pneumoniae* and has the highest iron affinity as compared with other siderophores (Hsieh et al., 2008; Li et al., 2014). The entC gene encodes isochorismate synthetase, which plays a critical role in enterobactin synthesis (Liu et al., 1990; Raymond et al., 2003). Rcs phosphorelay is well-known for its function in regulating CPS, its role in iron acquisition system is unclear.

Excess iron is toxic for bacteria (Becker and Skaar, 2014). Ferric uptake regulator (Fur), is a transcriptional regulator that alters gene expression in response to iron availability, regulates iron homeostasis in many bacteria (Seo et al., 2014). In general, holo-Fur (Fur bound to Fe^2+^) represses gene expression, whereas apo-Fur (Fur not bound to Fe^2+^) de-represses gene expression (Stacy et al., 2016). In *Escherichia coli*, holo-Fur can directly repress regulation of *entC* (Brickman et al., 1990). Therefore, we postulated that holo-Fur can also function as a repressor of *entC* under iron-rich conditions in *K. pneumoniae*.

In this study, we explored how RcsAB and Fur coregulate *entC* under different iron conditions. Our results suggested an regulatory cascade in which Fur regulates the *rcsA* and *entC* promoters. This study provides new light on the regulons of RcsAB and the mechanisms controlling iron acquisition in iron-repletion and iron-depletion.

## Materials and Methods

### Bacterial Strains, Plasmids, Primers, and Media

The bacterial strains and plasmids used in this study are listed in Table 1. The primers used in this study are listed in Table 2. Bacterial strains were routinely cultured aerobically at 37°C in Luria-Bertani (LB) broth or on LB agar plates with antibiotics added as required at the following concentrations: kanamycin, 50 μg/ml; chloramphenicol, 35 μg/ml; ampicillin, 100 μg/ml. Bacterial growth was monitored by measuring the optical density of the cultures at a wavelength of 600 nm (OD_600_).

**Table 1:** Bacterial strains and plasmids used in this study.

**Strains or plasmids**	**Relevant genotype or phenotype**	**Source or reference**
***K. pneumonia***		
NTUH-K2044	K1 serotype	Fang et al., 2004
Kp:*ΔrcsA*	K2044 with deletion of *rcsA*	This study
Kp:*ΔrcsB*	K2044 with deletion of *rcsB*	This study
Kp:*ΔrcsAB*	K2044 with deletion of *rcsA, rcsB*	This study
Kp:*Δfur*	K2044 with deletion of *fur*	This study
Kp:*cΔrcsA*	Kp:*ΔrcsA* complemented with *rcsA*	This study
Kp:*cΔrcsB*	Kp:*ΔrcsB* complemented with *rcsB*	This study
Kp:*ΔrcsAB/cΔrcsA*	Kp:*ΔrcsAB* complemented with *rcsA*	This study
Kp:*ΔrcsAB/cΔrcsB*	Kp:*ΔrcsAB* complemented with *rcsB*	This study
CCW01	NTUH-K2044 *ΔlacZ* strain	Wu et al., 2010
CCW01:*ΔrcsA*	CCW01 with deletion of *rcsA*	This study
CCW01:*ΔrcsB*	CCW01 with deletion of *rcsB*	This study
CCW01:*ΔrcsAB*	CCW01 with deletion of *rcsA, rcsB*	This study
CCW01/p*lacZ15-entC*	CCW01 complemented with *KP1_entC*	This study
CCW01:*ΔrcsA*/p*lacZ15-entC*	CCW01:*ΔrcsA* complemented with *KP1_entC*	This study
CCW01:*ΔrcsB*/p*lacZ15-entC*	CCW01:*ΔrcsB* complemented with *KP1_entC*	This study
CCW01:*ΔrcsAB*/p*lacZ15-entC*	CCW01:*ΔrcsAB* complemented with *KP1_entC*	This study
***E. coli***		
DH5α	Cloning host	Grant et al., 1990
BL21(DE3)	Express protein	Studier and Moffatt, 1986
**Plasmids**		
pKO3-km	Km^r^, suicide vector	Pan et al., 2008
pKO3-km-*rcsA*+	Km^r^, suicide vector for *rcsA* deletion	This study
pKO3-km-*rcsB*+	Km^r^, suicide vector for *rcsB* deletion	This study
pKO3-km-*fur*	Km^r^, suicide vector for *fur* deletion	This study
pBAD33	Cm^r^, cloning vector	Laboratory stock
pBAD33-*rcsA*	Cm^r^, cloning vector containing *rcsA*	This study
pBAD33-*rcsB*	Cm^r^, cloning vector containing *rcsB*	This study
p*lacZ*15	Cm^r^, promoter selection vector, *lacZ*+	Wu et al., 2010
p*lacZ*15-p*entC*	Cm^r^, *entC* promoter fused with *lacZ* reproter	This study
pET-28a	Km^r^, protein expression vector	Novagen
pET-28a- *rcsB*	Km^r^, pET-28a containing *rcsB*	This study
pET-28a- *fur*	Km^r^, pET-28a containing *fur*	This study
pMAL-C5X	Am^r^, protein expression vector	NEB
pMAL-C5X-*rcsA*	Am^r^, pMAL-C5X containing *rcsA*	This study
pMD19-T	Am^r^, cloning vector	TaKaRa
pMD19-T-*entC*-RcsAB	Am^r^, cloning vector containing putative RcsAB binding region of *entC* promoter	This study
pMD19-T-*entC*-Fur	Am^r^, cloning vector containing putative Fur binding region of *entC* promoter	This study

**Table 2:** Oligonucleotide used in this study.

**Primers**	**Sequence (5^**′**^-3^**′**^)**
**Gene deletions**	
*KP1_3552-rcsA*-A	GTATGCGGCCGCTTGGTGACAATCAGGCTGG
*KP1_3552-rcsA*-B	GAGGTGATGTTTTCGGTCAGGACCCATCCTCATTCAACAC
*KP1_3552-rcsA*-C	GTGTTGATTGAGGATGGGTCCTGACCGAAAACATCACCTC
*KP1_3552-rcsA*-D	GTATGCGGCCGCTAACGGTTGGCTTCACTGG
*KP1_3872-rcsB*-A	GTATGCGGCCGCTATCGTCCTGCTGGATGTG
*KP1_3872-rcsB*-B	CAGCGAGACGGAAGAGAGGTAGTGACTTACGAATACCGAACAG
*KP1_3872-rcsB*-C	CTGTTCGGTATTCGTAAGTCACTACCTCTCTTCCGTCTCGCTG
*KP1_3872-rcsB*-D	GTATGCGGCCGCATGGTCATCTTCTGTCGCC
*KP1_1659-fur*-A	GTATGCGGCCGCATCCCGACCTGGTACTAC
*KP1_1659-fur*-B	GTCGTGGGCGTGTTCGTCGCGGAATCTGTCCTGTTG
*KP1_1659-fur*-C	CAACAGGACAGATTCCGCGACGAACACGCCCACGAC
*KP1_1659-fur*-D	GTATGCGGCCGCTCCGCAGCAATAATACGA
**Complementation of mutant**	
*KP1_3552-rcsA*-HB-*KpnI*-F	TCCGGTACCAGGAGG*AATTCACC*ATGTCAACG ATGATTATGGA
*KP1_3552-rcsA*-HB-*Sal*I-R	ACGCGTCGACTCAGCGCATATTTACCTG
*KP1_3872-rcsB*-HB-*Kpn*I-F	TCCGGTACCAGGAGG*AATTCACC*ATGAACACT ATGAACGTAATT
*KP1_3872-rcsB*-HB-*Sal*I-R	ACGCGTCGACTTSCTCTTTGTCCGTCG
*KP1_1659-fur*-HB- *KpnI*-F	CGGGGTACCAGGAGG*AATTCACC*ATGAC TGACAACAATACC
*KP1_1659-fur*-HB-*Hind*III-R	CCCAAGCTTTTATTTTTCCACCGC
**Protein expression**	
*KP1_3552-rcsA-Sal*I-F	ACGCGTCGACATGTAAACGATGATTATGGATT
*KP1_3552-rcsA-BamH*I-R	CGCGGATCCTCAGCGCATATTTACCTGAA
*KP1_3872-rcsB*- *BamH*I-F	CGCGGATCCATGAACACTATGAACGTAATTATT
*KP1_3872-rcsB*- *Sal*I-R	ACGCGTCGACTTACTCTTTGTCCGTCGC
*KP1_1659-fur- BamH*I-F	CGCGGATCCATGACTGACAACAATACCG
*KP1_1659-fur*- *Hind*III-R	CCCAAGCTTTTATTTTTCCACCGCGTCG
**RT-qPCR**	
*KP1_entC-*RT-F	GTGCTATCAAGGCTTATCG
*KP1_entC-*RT-R	AGTGCTGTCCTTCTTTACG
*KP1_rcsA-*RT-F	CACCAGTGTAGGGCAGTT
*KP1_rcsA-*RT-R	GTGATGTTTTCGGTCAGC
*KP1_rho-*RT-F	AACTACGACAAGCCGGAAAA
*KP1_rho-*RT-R	ACCGTTACCACGCTCCATAC
**EMSA**	
*KP1_entC*-EMSA-RcsAB-F	GCTGGGTGAGCAGGGTTT
*KP1_entC*-EMSA-RcsAB-R	CGGGTCGGTTTCTTTACATC
*KP1_entC*-EMSA-Fur -F	GGTATGCGTCCCGTAGC
*KP1_entC*-EMSA-Fur -R	ACCTCCATCGCCTCCAG
*KP1_rcsA*-EMSA-Fur -F	CATCATTCATCCCACAAG
*KP1_rcsA*-EMSA-Fur -R	AAGATAACAAACAGCGTC
*KP1_16S*-EMSA-F	CGGTCTGTCAAGTCGGATGTG
*KP1_16S*-EMSA-R	CGGAAGCCACGCCTCAAG
**DNase I footprinting**	
*KP1_entC*-FP-RcsAB-F	CGTCACGCTGGTGGAGACAATG
*KP1_entC*-FP-RcsAB-R	GGTATTGCCCGCCATGTCAACC
*KP1_entC*-FP-Fur-F	TTGAAAGGTGATAAATGC
*KP1_entC*-FP-Fur-R	AGTGGTAAAACTGCGGTA
***lacZ*** **fusion**	
*KP1_entC*- *lacZ*-F	CGGGATCCGGCTCTGGCCGTTCAGAC
*KP1_entC*- *lacZ*-R	GAAGATCTGTGAGAAGCGACGGAAGC

*Amplification of the KP1_3552-rcsA, KP1_3872-rcsB, and KP1_1659-fur coding regions together with a ribosome binding site (underline) consensus sequence, AGGAGG, and a spacer, AATTCACC (italic)*.

### Construction of Gene Deletion and Complementation Strains

The mutants Kp-Δ*rcsA*, Kp-Δ*rcsB*, Kp-Δ*rcsAB*, and Kp-Δ*fur* were constructed as previously described (Peng et al., 2018; Su et al., 2018). Gene deletion was done by allelic replacement. In brief, the upstream and downstream flanking regions of target gene fragments were amplified, purified, fused, and cloned into the temperature-sensitive suicide vector pKO3-Km. The resulting plasmid was transformed into NTUH-K2044 by electroporation. After the recombinant plasmid was integrated (at 30°C) and excised (at 43°C), the mutants Kp:Δ*rcsA*, Kp:Δ*rcsB*, Kp:Δ*rcsAB*, and Kp:Δ*fur* were constructed and further verified by PCR and DNA sequencing.

For complementation experiments, the amplified DNA fragments were ligated to pBAD33. The recombinant plasmids were introduced into the mutant strains. The complementation strains were selected with chloramphenicol on LB agar plates and verified by PCR.

### Chrome Azurol S (CAS) Assay

The CAS assay described by Schwyn and Neilands was used to check the siderophores from bacteria (Schwyn and Neilands, 1987). Briefly, bacteria were inoculated into MM9 minimal medium (which contained the following components per liter: 100 ml of 10 × MM9 minimal medium [3 g of KH_2_PO_4_, 5 g NaCl, 1 g of NH_4_Cl], 30 ml of deferrated casamino acids, 10 ml of 20% glucose, 1 ml of 1 M MgCl_2_, 1 ml of 100 mM CaCl_2_, 30.24 g of PIPES, 6 g of NaOH) and cultured for 16 h. OD_600_ was read and siderophore levels were standardized by the OD_600_ measurements. The supernatants were collected, diluted with MM9 medium and subjected to the CAS assay with percent siderophore units calculated as previously described (Payne, 1994).

### *lacZ* Fusion and β-Galactosidase Assay

The putative promoter DNA region of *entC* was amplified by *KP1*_*entC-lacZ*-F/R and cloned into the p*lacZ*15 plasmid that harbors a promoterless *lacZ* reporter gene and transferred into *K. pneumoniae* NTUH-K2044Δ*lacZ* strain CCW01 and the deletion mutants. A single colony was inoculated into LB with or without 2,2-dipyridyl (Dip) and grown to logarithmic phase. Cells grown as described above were assayed for β-galactosidase activity (Luo et al., 2017), and the units of activity were calculated as described by Miller. Every sample was tested in triplicate, and the assay was repeated in at least three independent experiments.

### Reverse Transcription Quantitative Real-Time PCR (RT-qPCR)

NTUH-K2044, Kp:Δ*rcsA*, Kp:Δ*rcsB*, Kp:Δ*rcsAB*, and Kp:Δ*fur* were inoculated into LB liquid medium. After overnight growth, NTUH-K2044, Kp:Δ*rcsA*, Kp:Δ*rcsB*, and Kp:Δ*rcsAB* were diluted 1:100 in 15 ml of fresh medium with Dip (250 μM final concentration). NTUH-K2044 and Kp:Δ*fur* were diluted 1:1,000 in 15 ml of fresh medium with FeSO_4_ (100 μM final concentration). When the strains grew to logarithmic phase, total RNA was extracted using the TIANGEN RNAprep Pure Cell/Bacteria Kit following the manufacture's protocol. RNA integrity was analyzed by agarose gel electrophoresis, and RNA purity and concentration were calculated by measuring the optical density of the samples at 260 and 280 nm using a NanoDrop 2000 UV-Vis spectrophotometer (Thermo Scientific). Then, the RNA was converted to single-stranded cDNA using the PrimeScript™ RT Reagent Kit. Real-time PCR was carried out using a LightCycler® system. Relative gene expression was quantified using the Ct value-based method with *rho* (Gomes et al., 2018) rRNA as the internal control.

### Protein Expression and Purification

RcsA and RcsB were expressed and purified as previously described (Peng et al., 2018). Briefly, The entire coding regions of *rcsA* and *rcsB* were cloned into the pMAL-C5X and pET-28a, respectively. Then, the resulting plasmids were transformed into *E. coli* BL21 (DE3) cells, and the recombinant proteins MBP-RcsA and His_6_-RcsB were overexpressed under induction by isopropyl β-D-thiogalactopyranoside (IPTG). The cells were lysed by supersonication, and the proteins were purified by column chromatography and dialyzed.

Purification of the His_6_-Fur fusion protein was carried out as previous study described (Gao et al., 2008). The recombinant plasmid, pET-28a-*fur*, was transformed into *E. coli* BL21 (DE3) cells. The culture was grown at 37°C in LB medium overnight and then was transferred into 500 ml LB medium. The His_6_-Fur was induced with 1 mM IPTG at OD_600_ of 0.6 and expressed for 4 h at 30°C before the cells were harvested. The pellet was suspended in 15 ml buffer A (50 mM NaH_2_PO_4_, 300 mM NaCl, 10 mM imidazole, 10% glycerol, pH 8.0). The cells were lysed with sonication and centrifuged at 4°C. The supernatant was loaded onto a nickel column, and the column was washed with a gradient of 20–40 mM imidazole prepared in buffer A, respectively. The His_6_-Fur was eluted using buffer A with 250 mM imidazole. His_6_-Fur was further dialyzed in buffer B (10 mM NaH_2_PO_4_, 40 mM Na_2_HPO_4_, 145 mM NaCl, 30% glycerol) at 4°C. The MBP-RcsA, His_6_-RcsB, and His_6_-Fur were detected by sodium dodecyl sulfate-polyacrylamide gel electrophoresis (SDS-PAGE).

### Electrophoretic Mobility Shift Assay (EMSA)

The putative promoter regions of the *entC* gene were amplified by PCR using primers *KP1*_*entC*-EMSA-RcsAB-F/R and *KP1*_*entC*-EMSA-Fur-F/R that listed in Table 2. Mix 25 mM fresh acetyl phosphate, His_6_-RcsB and binding buffer (50 mM Tris-HCl, 750 mM KCl, 0.5 mM DTT, 0.5 mM EDTA) and incubate at 37°C for 30 min to phosphorylae the His_6_-RcsB. The target *entC* promoter DNA (20 ng) was mixed with increasing amounts of MBP-RcsA or phosphorylated His_6_-RcsB. After incubation at 37°C for 30 min, the samples were analyzed by 4% (w/v) PAGE in 0.5 × TBE buffer (45 mM Tris-HCl, 45 mM boric acid, 1 mM EDTA).

EMSAs for the *entC* and *rcsA* promoters by His_6_-Fur, DNA were incubated with purified His_6_-Fur in a 10-μl solution containing 50 mM Tris-HCl, 5 mM MgCl_2_, 250 mM KCl, 20% glycerol, 2.5 mM dithiothreitol, 0.25 mg/ml BSA, 500 μM MnCl_2_ at 37°C for 20 min. Then, the samples were examined by separation on a native 4% (w/v) polyacrylamide gels in 0.5 × TB buffer (45 mM Tris-HCl, 45 mM boric acid). A constant voltage of 150 V was applied to all gels for 2 h at 4°C. After staining with SYBR Green EMSA stain (Invitrogen), the gel was examined with a UV transilluminator.

### DNase I Footprinting

The target DNA fragment of the promoter DNA region was PCR amplified using the primers M13F-47 (FAM) and M13R-48 with DNA polymerase premix from the constructed plasmid for preparation of fluorescent FAM labeled probes. DNase I footprinting assays were performed in a manner similar to the method described by Wang et al. (2012). DNase I footprinting assay for *entC* promoter by RcsAB, porbes were incubated with 0, 1, 3 μg of His_6_-RcsB for 30 min at 37°C. Then MBP-RcsA was added and this mixture incubated for 30 min at 37°C. DNase I footprinting assay for *entC* promoter by Fur, probes were incubated with 0, 0.5, 1 μg of His_6_-Fur for 30 min at 37°C. After adding DNase I (Promega), samples were extracted with phenol/chloroform, then precipitated with ethanol. Pellets were dissolved in 30 μl MiniQ water. The preparation of DNA ladder, capillary electrophoresis and data analysis were the same as described before (Wang et al., 2012), except that the GeneScan-LIZ600 size standard (Applied Biosystems) was used.

### Statistical Analysis

All experiments were performed at least three times. Results were presented as means ± standard deviation (SD). GraphPad Prism 7.0 software (GraphPad Software, Inc, La Jolla, CA, USA) was used for statistical. Statistical significance was determined using one-way ANOVA for multiple comparisons and Student's *t*-test for comparing two groups. Asterisk indicate *P* values (^*^P < 0.05).

## Results

### RcsAB Affects the Iron Acquisition System

**Figure 1:**
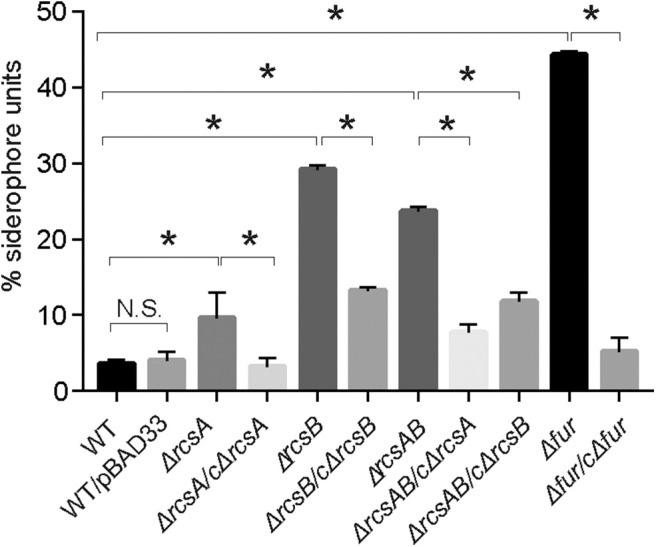
The *K. pneumoniae* iron acquisition system is modulated by RcsAB and Fur. CAS assay for *K. pneumoniae* NTUH-K2044 wild type, wild type with empty plasmid, mutants, and complement strains were assessed as described in Material and Methods. Siderophores secreted from strains removed iron from dye complex giving rise to a reduction in blue color of the solution. Measure the absorbance (A_630_) for loss of blue color. Results were the means of biological triplicates plus standard deviations. Bar graph showed percent siderophore units, calculated as [(Ar – As) / Ar] × 100, where Ar is the absorbance of MM9/CAS solution and As is the sample absorbance. And MM9 can be used as a blank. The percent siderophore units of WT was compared with mutants and the percent siderophore units of mutants were compared with its complement strains. *P* values were calculated by one-way ANOVA. *P < 0.05. Shown are the averages ± standard deviation (SD) from three independent experiments.

RcsAB and Fur are transcriptional regulators that can regulate various virulence factors in NTUH-K2044. And iron acquisition system is an important virulence factor. To analyze whether RcsAB and Fur affect iron acquisition in NTUH-K2044, an *in vitro* Chrome azurol S (CAS) assay examining the secretion of siderophores was performed. As shown in Figure 1, no statistical difference was found in WT and WT with empty plasmid. Siderophore secretion by Kp:Δ*rcsA*, Kp:Δ*rcsB*, Kp:Δ*rcsAB*, and Kp:Δ*fur* were increased by 3, 8, 7, and 12-fold relative to WT, respectively. Complementation with plasmid-encoded *rcsA, rcsB*, and *fur* led to a decrease in secretion of siderophore relative to the mutant strains. Collectively, these results indicated that the iron acquisition in NTUH-K2044 was influenced by RcsAB and Fur.

### Iron Limitation Enhances the Activity of the *entC* Promoter

**Figure 2:**
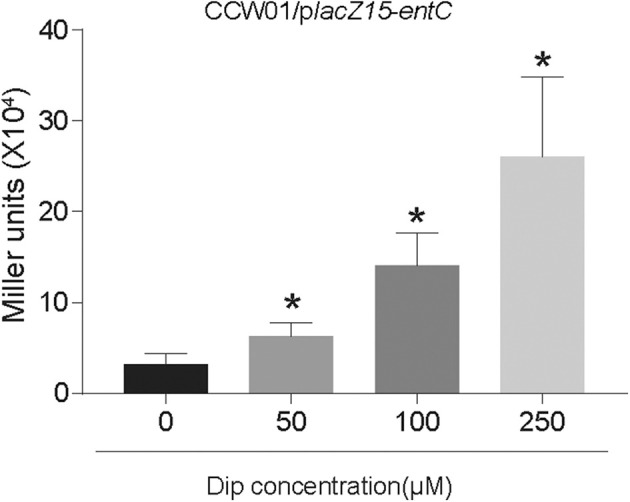
Iron starvation enhanced the activity of the *entC* promoter. The putative promoter region of *entC* was cloned into the p*lacZ*15 plasmid and then introduced into CCW01 to measure β-galactosidase activity (Miller units) in increasing amounts of Dip. *P < 0.05, compared with the 0 μM Dip group.

Ent, a siderophore, is enriched under iron restriction. The *entC* participates in the synthesis of Ent. To examine whether the restriction of environmental iron increases *entC* expression, we cloned the putative promoter regions upstream of *entC* into the *lacZ* reporter plasmid and transformed the constructs into CCW01. And CCW01/p*lacZ*15 was used as a control. For iron depletion, increasing amounts of the iron chelator 2,2-dipyridyl (Dip) were added to LB medium. Figure 2 shows that as the concentration of Dip increased, the activity of the *entC* promoter increased. The addition of 50, 100, and 250 μM Dip to the medium increased *entC* promoter activity by ~2, 4, and 8-fold, respectively. There were no statistical differences of β-galactosidase from CCW01/p*lacZ*15 under different concentrations of Dip (data not shown), suggesting unspecific regulation by iron is unlikely. The promoter activity of *entC* was activated under iron-limited conditions.

### RcsAB and Fur Coregulate *entC* Expression

**Figure 3:**
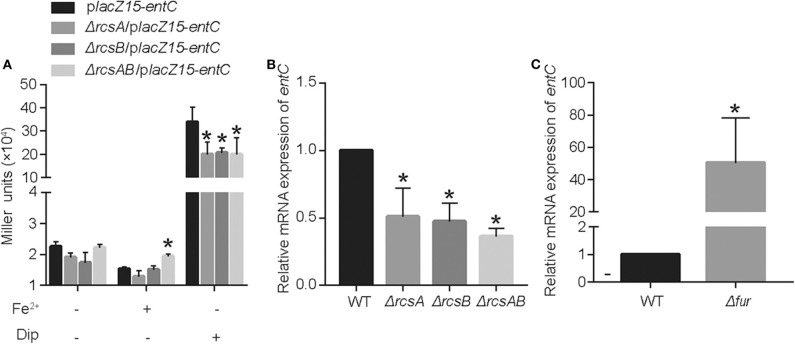
RcsAB and Fur coregulate *entC* expression under different iron levels. **(A)** The promoter-*lacZ* fusion for *entC* was transformed into the CCW01, CCW01:Δ*rcsA*, CCW01:Δ*rcsB*, and CCW01:Δ*rcsAB* that were then grown in LB, LB with 100 μM FeSO_4_ and LB with 250 μM Dip. **(B)** When 250 μM Dip added to LB. *entC* mRNA levels in WT, Kp:Δ*rcsA*, Kp:Δ*rcsB*, and Kp:Δ*rcsAB* were measured in iron-restricted conditions via RT-qPCR analysis. One-way ANOVA was performed to determine statistically significant differences between each strain and the WT. **(C)** When 100 μM FeSO_4_ added to LB, WT, and Kp:Δ*fur* were examined for *entC* expression under iron-replete conditions by RT-qPCR analysis. *P < 0.05, compared with the WT. Error bars are standard deviation.

RcsAB and Fur are predicted and have been shown to impact the iron acquisition system. We sought to examine how these proteins regulated *entC* expression according to the level of iron in the environment. In LB medium, there are no statistical differences among CCW01/p*lacZ15-entC*, CCW01:Δ*rcsA*/p*lacZ15-entC*, CCW01:Δ*rcsB*/p*lacZ15-entC*, and CCW01:Δ*rcsAB*/p*lacZ15-entC*. After adding FeSO_4_ to LB, bacteria were in iron replete conditions and expression from *entC* promoter was not altered in CCW01:Δ*rcsA*/p*lacZ15-entC*, CCW01:Δ*rcsB*/p*lacZ15-entC*, although the miller units of CCW01:Δ*rcsAB*/p*lacZ15-entC* were higher than CCW01/p*lacZ15-entC*. However, when Dip added to LB, bacteria were in iron-restricted conditions. CCW01:Δ*rcsA*/p*lacZ15-entC*, CCW01:Δ*rcsB*/p*lacZ15-entC*, and CCW01:Δ*rcsAB*/p*lacZ15-entC* all led to 2–fold less β-galactosidase activity relative to CCW01/p*lacZ15-entC* (Figure 3A). These results demonstrated that RcsAB positively regulated *entC* transcription under iron deficient conditions.

To verify further that RcsAB regulate *entC* expression under iron restricted conditions, we determined the mRNA levels of *entC* in WT, Kp:Δ*rcsA*, Kp:Δ*rcsB*, and Kp:Δ*rcsAB* by RT-qPCR. When LB medium was supplemented with 250 μM Dip, transcription of *entC* by Kp:Δ*rcsA*, Kp:Δ*rcsB*, and Kp:Δ*rcsAB* were decreased by 1.4, 1.8, and 2.3-fold relative to WT, respectively (Figure 3B). On the other hand, after adding FeSO_4_ to LB, deletion of *fur* resulted in a dramatic increase in expression of *entC* mRNA (Figure 3C). These data suggest that RcsAB and Fur coregulated *entC* in response to iron availability.

**Figure 4:**
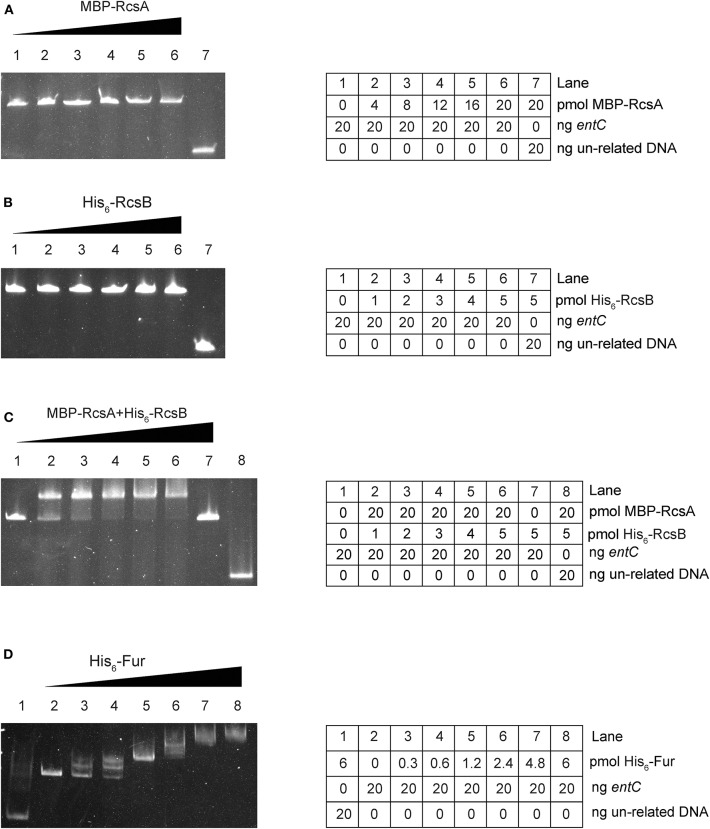
RcsAB and Fur bind the *entC* promoter. The DNA binding capacities were evaluated by EMSAs. The promoter DNA fragments of *entC* were incubated with increasing amounts of purified MBP-RcsA **(A)**, His_6_-RcsB **(B)**, His_6_-RcsB mixed with MBP-RcsA **(C)**, and His_6_-Fur **(D)** and then subjected to polyacrylamide gel electrophoresis.

To investigate further whether *entC* served as a direct target of RcsAB and Fur, EMSAs were performed. MBP-RcsA, His_6_-RcsB, His_6_-RcsB mixed with 20 pmol MBP-RcsA and His_6_-Fur were subjected to EMSAs with the purified whole promoter DNA region of *entC*. Neither MBP-RcsA nor His_6_-RcsB binded to the *entC* upstream DNA (Figures 4A,B). However, His_6_-RcsB mixed with 20 pmol MBP-RcsA and His_6_-Fur could bind to the putative *entC* promoter DNA fragment (Figures 4C,D).

**Figure 5:**
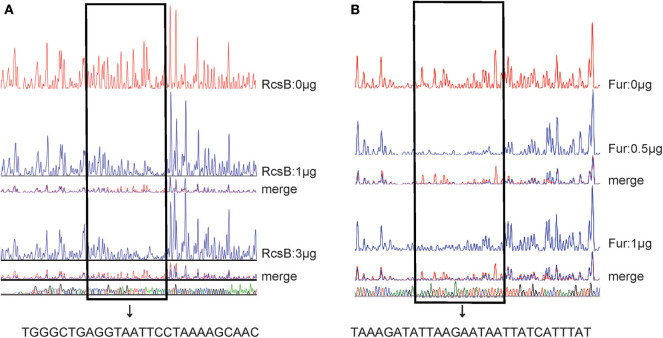
DNase I footprints of RcsAB and Fur at the *entC* promoter. The promoter DNA regions of *entC* were labeled with FAM and incubated with increasing amounts of purified His_6_-RcsB (0, 1, 3 μg) **(A)** and His_6_-Fur **(B)** (0, 0.5, 1 μg). The footprint regions are boxed within lines and marked.

**Figure 6:**
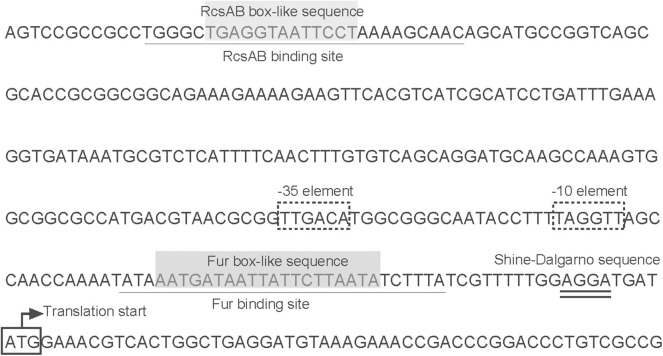
Organization of *entC* promoter-proximal DNA regions. The DNA sequences were derived from NTUH-K2044. Shown are the translation start sites,−10 and−35 elements, Shine-Dalgamo sequence, RcsAB-binding site, RcsAB box-like sequence, Fur-binding site and Fur box-like sequence.

As further determined by DNase I footprinting (Figures 5A,B), both His_6_-RcsB mixed with MBP-RcsA and His_6_-Fur protected the DNA region upstream of *entC*, covering ~27 bases and 29 bases, respectively. Besides, the protected regions corresponded to the predicted binding sites for these proteins as indicated in Figure 6. The *entC* promoter was constructed with translation start site, core promoter−10 and−35 elements (Brickman et al., 1990), Shine-Dalgamo sequence, RcsAB box-like sequences, RcsAB sites, Fur box-like sequences, and Fur sites (Figure 6). Taken together, RcsAB and Fur directly coregulate *entC* expression.

### Fur Directly Represses *rcsA* Expression Under Iron Repletion

**Figure 7:**
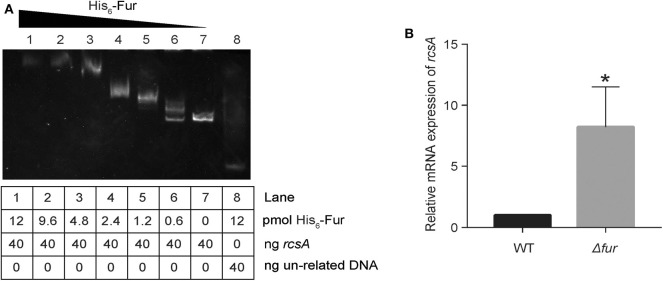
Fur directly represses *rcsA*. **(A)** The DNA binding capacity of Fur was evaluated by EMSA with *rcsA*. **(B)** Relative mRNA expression of *rcsA* in WT and Kp:Δ*fur* was assayed by RT-qPCR in an iron-rich environment. *P < 0.05, compared with the WT.

RcsA, an auxiliary activator protein, acts with RcsB as a transcription regulator. In *K. pneumoniae* CG43, the expression of *rcsA* is reportedly regulated by Fur (Lin et al., 2011). Due to the heterogeneity of *K. pneumoniae* genomes, we tested whether Fur repressed *rcsA* expression in a direct way in NTUH-K2044. A putative Fur-binding box was located at the translation start site of *rcsA* in NTUH-K2044. Thus, to verify whether Fur could bind to the putative promoter regions of *rcsA*, we performed an EMSA. As shown in Figure 7A, His_6_-Fur was able to bind to the regions.

Additionally, when iron is abundant in the environment, holo-Fur represses the regulons (Seo et al., 2014). To confirm that *rcsA* expression was indeed increased in Kp: Δ*fur* under iron repletion, we measured the transcript levels of *rcsA* via RT-qPCR. As detailed in Figure 7B, we observed an 8-fold (*p* < 0.05) increase of *rcsA* transcript levels in Kp: Δ*fur*. These data confirm that Fur negatively regulates *rcsA* expression under iron repletion and represses the biosynthesis of RcsAB in NTUH-K2044, which indirectly downregulates *entC* expression. Furthermore, Fur could repress *entC* in a direct way.

## Discussion

Iron acquisition system is important for virulence of *K. pneumoniae*. Numerous transcriptional regulators are involved in the process of iron uptake and metabolism, such as Fur (Gao et al., 2008; Seo et al., 2014). Dorman and colleagues (Dorman et al., 2018) found Rcs phosphorelay system is a major node for transcriptional control. Intriguingly, the identification of the RcsAB box upstream of *entC* that plays a crucial role in the biosynthesis of Ent in *K. pneumoniae* suggests that RcsAB may also involve in the regulation of iron acquisition system. Our data suggested that RcsAB contribute to not only CPS and mobility but also the iron acquisition system (Figure 1).

The Rcs phosphorelay is a signal transduction system, and the phosphorylation and dephosphorylation of RcsC, RcsD, and RcsB were affected by environmental signals, such as overproduction of DjlA, YmgA, and YmgB; the presence of a solid surface; osmotic shock; acid shock; and growth at low temperature in the presence of glucose and 1 mM zinc (Sledjeski and Gottesman, 1996; Kelley and Georgopoulos, 1997; Ferrieres and Clarke, 2003; Hagiwara et al., 2003; Kannan et al., 2008; Tschowri et al., 2009). Therefore, we hypothesized that iron status may activate the Rcs system, which has never been reported in any organism.

**Figure 8:**
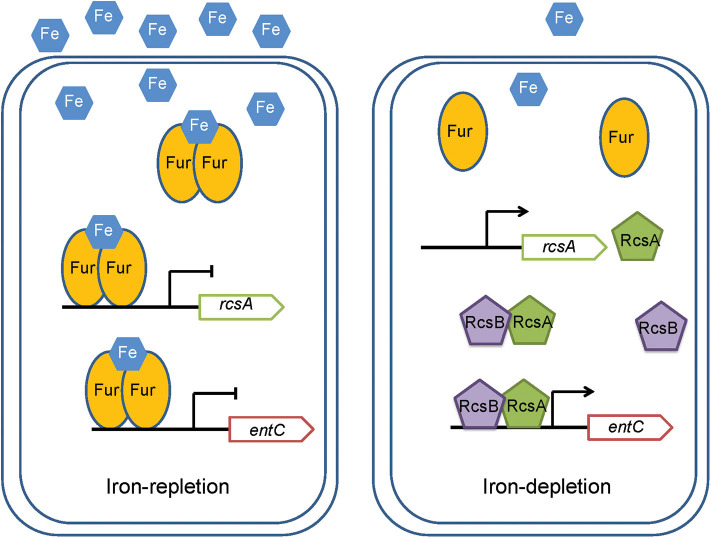
A model for the regulation by RcsAB and Fur of *rcsA* and *entC* in *K. pneumoniae*. Under iron-replete conditions, apo-Fur combines with Fe^2+^ to form holo-Fur, which strongly represses *rcsA* and *entC*. Under iron depletion, upon derepression by apo-Fur of *rcsA* and *entC* and activation of RcsAB, the transcription of *entC* increased.

RcsA is an unstable positive regulator required for the synthesis of CPS (Stout et al., 1991). And Fur regulate the *rcsA* to control the expression of CPS in *K. pneumoniae* CG43 (Lin et al., 2011). Our study suggested a regulatory cascade exists under iron repletion in which Fur controls *rcsA* expression. The reduction in *rcsA* levels appears to have impacts on the synthesis of RcsAB and thus affects the expression of *entC*. RcsB alone can regulate expression of many genes. When act as combination with other regulators, such as RcsA, RcsB can regulate the expression of wider spectrum of genes. Our result suggested that RcsB or RcsA alone is unable to regulate *entC*. Instead, it appears that these proteins form a complex that can bind to the promoter of *entC* (Figure 4). Hence, our study suggest a model in which when iron is restricted, Fur repression of *rcsA* is relieved. RcsA then binds with RcsB to activate *entC* expression (Figure 8).

Fur represses the transcription of *entC* when iron is sufficient, while RcsAB promotes *entC* expression in the absence of iron. However, under iron repletion, according to the results of the *lacZ* fusion assays, the β-galactosidase activity (Miller units) of *entC* in CCW01:Δ*rcsAB* was higher than that in CCW01/p*lacZ*15-p*entC* (Figure 3A). It is possible that the decrease in Ent expression is compensated by other transcriptional regulators due to the lack of RcsAB even in the presence of iron, as the iron acquisition system is regulated by multiple transcriptional regulators. As an example, some studies reported that cyclic AMP receptor protein (CRP) can also regulate *entC* in *E. coli* (Zhang et al., 2005; Seo et al., 2014). CRP is a global transcriptional regulator and regulates virulence-related gene expression (Xue et al., 2016). It is likely that CRP may regulate the iron acquisition system by regulating *entC* in *K. pneumoniae*.

Fur was not considered to be a regulator of *rcsB* as supported by Lin et al. (2011) and our preliminary result using RT-qPCR (data not shown). RcsB is the response regulator in Rcs phosphorelay. RcsB either alone or with auxiliary protein, such as RcsA, BglJ, MatA, could regulate genes expression. Importantly, RcsB serves as an essential partner. Hence, when the expression of RcsA or other auxiliary proteins have been affected by other transcriptional regulators, such as Fur, the regulons which regulated by complex proteins also can be affected. However, RcsB-dependent regulons cannot be affected.

Given that untimely expression of Ent under iron repletion conditions likely provides a detrimental energy burden, Fur represses *entC* transcription. Ent can be neutralized by the host-secreted molecule lipocalin-2 (Raymond et al., 2003). Hence, the expression of *entC* was reduced by Fur, which could protect *K. pneumoniae* from attacking by host immune system. Additionally, repression of *rcsA* expression by Fur give rise to a decrease of RcsAB, which negatively regulate *fim* gene cluster expression (Su et al., 2018). And fimbriae are important mediators of *K. pneumoniae* adhesion (Paczosa and Mecsas, 2016). Therefore, relatively high extracellular iron concentrations result in the upregulation of fimbriae, which is beneficial for adhesion and colonization of *K. pneumoniae*.

In summary, our study results suggested that RcsAB could modulate the iron acquisition system by directly regulating *entC* positively under iron starvation conditions, and Fur could directly repress *entC* under iron-rich conditions. Our study suggested a regulatory cascade by which Fur controls *rcsA* expression and the synthesis of RcsAB, and Fur also impacts *entC* expression. This study improves current understanding the mechanism of regulation of virulence factors by RcsAB in *K. pneumoniae* and the importance of the iron acquisition system in bacteria.

## Data Availability Statement

All datasets generated for this study are included in the article/supplementary material.

## Author Contributions

JQ contributed the conception. YL and LY designed the study. XL, LD, JZ, and KS performed the experiments. PL, QH, ZZ, DP, and LS analyzed the data. LY wrote the manuscript.

## Conflict of Interest

The authors declare that the research was conducted in the absence of any commercial or financial relationships that could be construed as a potential conflict of interest.
